# Clinical effectiveness of lumbosacral orthoses in chronic non-specific low back pain: a systematic review

**DOI:** 10.3389/fresc.2026.1857493

**Published:** 2026-06-08

**Authors:** Eray Yüceel, Tuğba Kuru Çolak, Aleyna Karahan, Ece Zeynep Saatçı, Burçin Akçay, Maksym Borysov, Hans-Rudolf Weiss

**Affiliations:** 1 Marmara University, Faculty of Health Sciences, Department of Physiotherapy and Rehabilitation, Istanbul, Türkiye; 2 Marmara University, Institute of Health Sciences, Department of Physiotherapy and Rehabilitation, Istanbul, Türkiye; 3 Dokuz Eylül University, Faculty of Physical Therapy and Rehabilitation, İzmir, Türkiye; 4 Orttech Plus Rehabilitation Service, Kharkiv, Ukraine; 5 Schroth Best Practice Academy, Neu-Bamberg, Germany

**Keywords:** brace, disability, low back pain, orthotic devices, pain, physical therapy modalities

## Abstract

**Introduction:**

Chronic non-specific low back pain (CLBP) is a common and disabling condition. Lumbosacral orthoses are used in conservative care, but their clinical benefit remains unclear. This systematic review evaluated the clinical effectiveness of lumbosacral orthoses in adults with CLBP.

**Methods:**

A search was conducted in PubMed, Scopus, Ovid, PEDro, and Web of Science up to July 2025. Randomized controlled trials (RCTs) investigating lumbar or lumbosacral orthoses in adults with CLBP were included. Methodological quality was assessed using the PEDro scale, and the level of evidence was classified descriptively using the Oxford Centre for Evidence-Based Medicine framework. The review followed the PRISMA 2020 guidelines.

**Results:**

Eleven RCTs involving 691 participants were included. The studies used different types of lumbosacral orthoses, including rigid, semi-rigid, soft, extensible, and inextensible designs. Intervention duration ranged from a single session to 12 months. Several studies reported improvements in pain or disability after lumbosacral orthosis use, especially in short-term intervention periods. The findings were not consistent across studies, and meta-analysis was not possible because of methodological heterogeneity. No study reported orthosis-induced muscle atrophy, but adverse effects, adherence, and patient tolerance were not consistently assessed. Objective neuromuscular outcomes and psychosocial measures were also limited.

**Discussion:**

Current evidence suggests that lumbosacral orthoses may be helpful for selected individuals with CLBP, especially when used as part of multimodal rehabilitation. Further high-quality RCTs with standardized protocols and longer follow-up are needed.

## 1 Introduction

Chronic low back pain (CLBP) is commonly defined as pain localized below the costal margin and above the inferior gluteal folds that persists for at least 12 weeks or 3 months (1). Low back pain (LBP) is highly prevalent, affecting most individuals at some point in their lives (2, 3). In 2020, approximately 619 million people globally—around one in 13—were affected by LBP, representing a 60% increase since 1990 (2, 4). Despite its widespread occurrence, significant gaps remain in clinical practice, with many treatments lacking a strong evidence base (2, 5).

The risk of CLBP ranges from 51% to 84%, increases with age, and is more prevalent in women (6). In patients with persistent symptoms, surgical treatment may be considered in selected cases; however, spinal surgery does not always result in satisfactory outcomes and may lead to failed back surgery syndrome (FBSS), particularly as spinal procedures become more common and complex in older adults (6). Therefore, in the absence of serious or progressive pathology requiring surgical intervention, non-surgical treatment strategies should be prioritized whenever possible (6, 7).

Current guidelines recommend a multimodal approach for CLBP, including physiotherapy and cognitive-behavioral or psychological interventions when appropriate (2). Within this conservative treatment framework, lumbar and lumbosacral orthoses are used to support posture, improve spinal alignment, reduce mechanical load, and relieve symptoms. However, their clinical effectiveness remains debated.

A sedentary lifestyle and altered spinal alignment have also been discussed as potential contributors to CLBP. Sedentary behavior is often associated with reduced lumbar lordosis and degenerative scoliosis in adults (8–12). Similarly, idiopathic scoliosis may involve sagittal profile disturbances, particularly in the thoracolumbar and lumbar regions (13). Previous studies have suggested that curve-specific braces, or braces designed to restore physiological lumbar lordosis, may be effective in selected patients with adult scoliosis and chronic low back pain (14, 15). Therefore, studies addressing scoliosis-related, lordosis-related, or posture-related CLBP were considered relevant when CLBP was the primary clinical focus and an orthosis was used as the intervention.

Directional diagnostics may help differentiate instability-related and postural CLBP and may support more targeted treatment approaches (16). Although some studies have reported positive findings for specific orthotic designs (16, 17), the clinical value of orthoses in CLBP remains debated. Therefore, further evaluation of targeted orthotic approaches in CLBP is needed.

A clear distinction between specific and non-specific low back pain is important. Low back pain is broadly classified as specific or non-specific depending on whether a definitive underlying cause can be identified. Non-specific low back pain is defined as pain not attributable to a recognizable specific pathology, such as infection, tumor, osteoporosis, fracture, structural deformity, inflammatory disorder, radicular syndrome, or cauda equina syndrome (18). In contrast, specific low back pain refers to cases in which an identifiable pathological mechanism can be established, such as nerve root involvement, radicular symptoms, or neurological deficits (2, 18). These diagnostic boundaries are important because the present review focuses on chronic non-specific low back pain rather than low back pain caused by identifiable specific pathology.

Despite the widespread use of lumbar and lumbosacral orthoses in clinical practice, evidence regarding their effectiveness in chronic non-specific low back pain remains inconclusive. Van Duijvenbode et al. (2008), in a comprehensive Cochrane review, concluded that there was no strong evidence that lumbar supports were more effective than other treatments or no treatment for non-specific low back pain (19). Although this review represented an important synthesis of the evidence available at that time, the evidence base is now outdated in light of more recent studies investigating diagnosis-specific orthotic designs, curve-specific braces, lordosis-restoring braces, and subgroup-targeted prescription strategies.

Based on this rationale, lumbosacral orthoses may have a role in the conservative management of CLBP. Therefore, this review aimed to qualitatively assess the existing evidence on the clinical effectiveness of lumbosacral orthoses in adults with CLBP.

## 2 Materials and methods

### 2.1 Search strategy

The search was conducted in the PubMed, Scopus, Ovid, PEDro, and Web of Science databases through remote access provided by the Marmara University Library and Documentation Directorate. The current literature was searched using the following search terms: (“Chronic Low Back Pain” (All Fields) OR “Persistent Low Back Pain” (All Fields) OR “Non-Specific Low Back Pain”(All Fields) OR “Lumbago” (All Fields)) AND (“Orthosis” (All Fields) OR “Back Brace” (All Fields) OR “Lumbar Support” (All Fields) OR “Spinal Orthosis” (All Fields) OR “Lumbosacral Orthosis” (All Fields) OR “Orthotic Devices” (Mesh) OR “Orthotic Devices” (All Fields) OR “Braces” (All Fields) OR “Braces” (Mesh) OR “Trunk Orthosis” (All Fields) OR “Corset” (All Fields)) AND (“Randomized Controlled Trial” OR “Randomised Controlled Trial” OR “RCT”) search strategy. Similar search terms or synonyms were also used in alternative databases. Only studies published in Turkish and English were considered. The search included relevant studies published up to July 2025. The search and reporting process followed the Preferred Reporting Items for Systematic Reviews and Meta-Analyses (PRISMA) checklist (20).

### 2.2 Eligibility criteria

Randomized controlled trials involving adult participants aged ≥18 years with chronic non-specific low back pain, defined as pain lasting ≥12 weeks and not attributable to a clearly identifiable specific pathology, were included. Eligible studies were required to investigate lumbar or lumbosacral orthosis-based interventions, either alone or in combination with other conservative treatments, and to compare these interventions with no orthotic intervention, physiotherapy or exercise alone, pharmacological treatment, alternative orthotic designs, or standard care.

Studies involving participants with acute or subacute low back pain only, or those evaluating preoperative or postoperative orthosis use, were excluded. Non-randomized study designs, including case reports, case series, cohort studies, retrospective or prospective observational studies, non-randomized controlled trials, and quasi-experimental studies, were also excluded. Conference abstracts, theses, and studies not available in English or Turkish were excluded.

For descriptive interpretation of the findings, outcomes assessed during or directly after a single orthosis application were considered immediate effects. Outcomes assessed after an intervention period of up to 12 weeks were considered short-term effects, whereas outcomes assessed after more than 12 weeks of orthosis use or follow-up were considered longer-term effects.

### 2.3 Study selection process

Following the removal of duplicate records, two researchers (EY and AK) independently screened the titles and abstracts of the identified articles. Potentially eligible studies were then assessed in full text. Disagreements regarding study eligibility were resolved by discussion and, when necessary, consultation with a third reviewer (TKÇ). The study selection process was reported in accordance with the PRISMA 2020 guidelines (20).

### 2.4 Evidence level classification

The Oxford Centre for Evidence-Based Medicine (OCEBM) 2011 Levels of Evidence framework was used to describe the evidence level of the included studies (21). This classification was applied after study selection and was not used as an inclusion or exclusion criterion. Because the review included only randomized controlled trials, the OCEBM framework was used descriptively to characterize the overall level of evidence rather than to grade the certainty of findings.

### 2.5 Assessment of methodological quality

The methodological quality of the included randomized controlled trials was independently assessed by two reviewers (AK and EZS) using the Physiotherapy Evidence Database (PEDro) scale (22). The PEDro scale is a validated and widely used tool for evaluating the internal validity and methodological rigor of clinical trials in physiotherapy. It consists of 11 items, of which 10 contribute to the total score, ranging from 0 to 10. The PEDro scale was used only to assess methodological quality and was not used to determine the level of evidence. In this review, studies scoring ≥6 were considered to be of high methodological quality, whereas those scoring <6 were classified as lower quality. Discrepancies between reviewers in PEDro scoring were resolved by consensus or through consultation with a third reviewer (EY).

## 3 Results

### 3.1 Descriptive data

**Figure 1:**
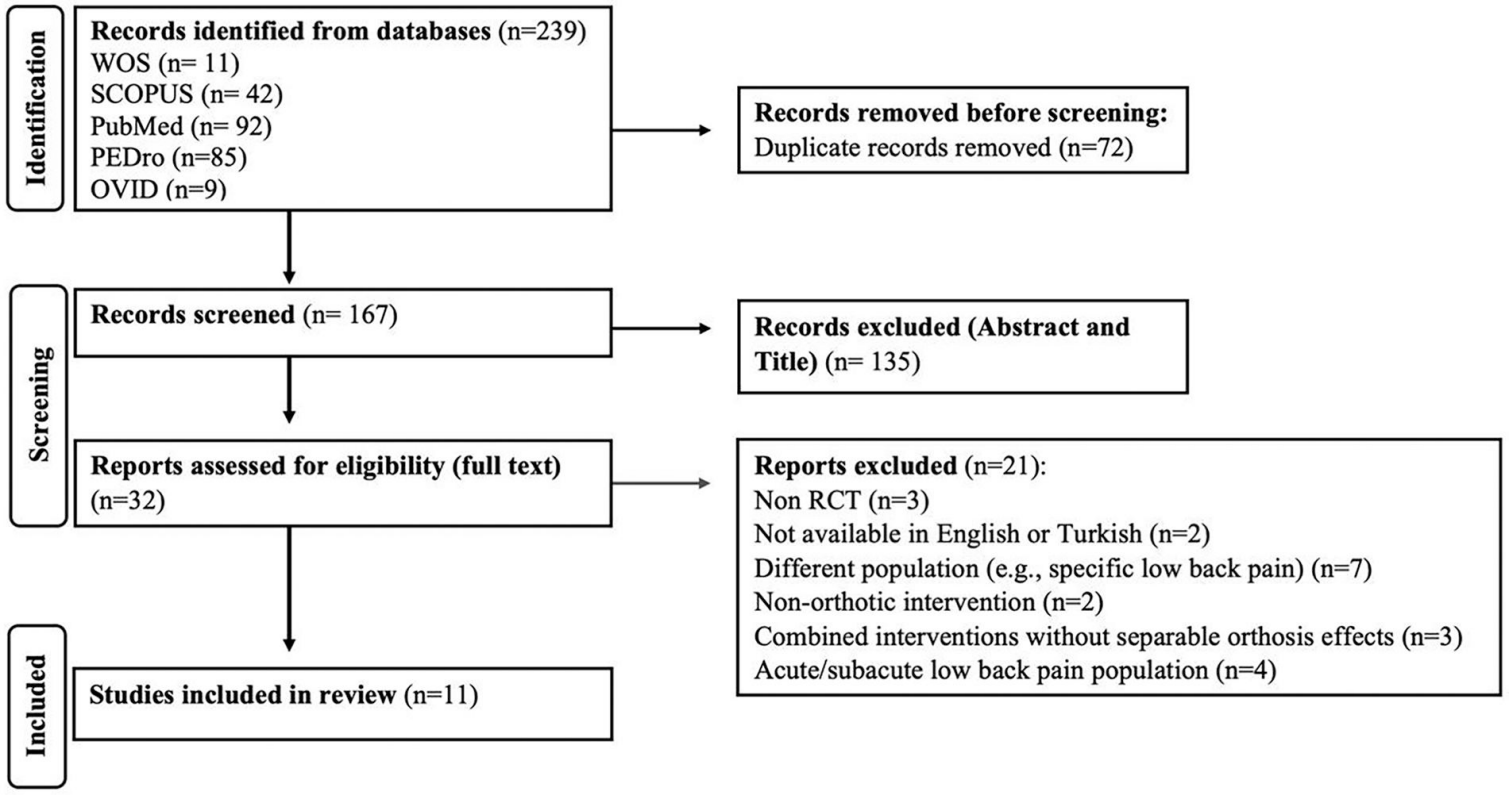
Flow chart of study evaluation and selection process according to PRISMA 2020.

As presented in the PRISMA 2020 flow diagram (Figure 1), two researchers identified 239 potentially relevant articles through five electronic databases. After the removal of 72 duplicate records, 167 titles and abstracts were independently screened, of which 135 were excluded for not meeting the eligibility criteria. Subsequently, 32 full-text articles were assessed for eligibility, resulting in the inclusion of 11 studies in the final systematic review (23–33).

The study characteristics are summarized in Table 1. The 11 studies that met the inclusion criteria were published between 2002 and 2022 and were conducted in five countries. Five studies were conducted in Iran (27, 29–31, 33) and three in Japan (24, 25, 28) (Table 2). All of the included studies are randomized controlled trials. Three studies conducted in Iran appear to have used the same sample group (27, 29, 30), but they investigated different outcome measures and were therefore included separately in the review.

**Table 1:** Characteristics of the studies in this review.

Author	Year	Country	Sample Size (Male/Female, Age)	Age	Intervention	Control Group	Outcomes
de Oliveira FCL. et al. (23)	2022	Canada	LSO: 20 (6/14, 29), Control Group: 20 (10/10, 29)	21–45	LSO	Abdominal Drawing Maneuver	COP Parameters
Hekmatfard M. et al. (33)	2021	Iran	Semirigid LSO: 12 (3/9, 31), Control Group 12 (3/9, 31)	18–50	Semirigid inextensible LSO + 10 physical therapy sessions for 3 weeks.	10 physical therapy sessions for 3 weeks.	Walking Complexity
Annaswamy TM. et al. (32)	2021	USA	Intervention Group: 25, Control Group: 34, Total Sample Size *n* = 59 (48/11, 48)	18–85	LO (Horizon 627) + Back School + Exercise Treatment	Back School + Exercise Treatment	Pain Disability Questionnaire, NPRS, Patient-Reported Outcome Measurement Information System, EuroQol 5-Dimension
Azadinia F. et al. (29)	2019	Iran	Intervention Group: 22 (9/13, 27), Control Group: 22 (8/14, 27)	20–55	Non-extensible LSO + routine physical therapy 8 sessions, twice a week for 4 weeks	Routine physical therapy 8 sessions, 2 sessions per week for 4 weeks	Postural Conditions and COP Parameters
Samani M. et al. (31)	2019	Iran	High pressure group: 13 (1/12, 37), Normal pressure group: 14 (3/11, 35), Control group: 15 (6/9, 34)	25–55	The normal pressure soft LSO High pressure (%50) soft LSO for 4 weeks + NSAID	NSAID	VAS, Modified Oswestry Disability Questionnaire, Isometric, isokinetic, concentric, eccentric strength and proprioception measurements in trunk flexor and extensor muscles, Electromyographic recording in trunk muscles.
Azadinia F. et al. (30)	2019	Iran	Intervention Group: 22 (9/13, 27), Control Group: 22 (8/14, 27)	20–55	Non-extensible LSO (QuikDraw Brace) + routine physical therapy twice a week for 4 weeks	Routine physical therapy twice a week for 4 weeks	Ultrasound measurement for muscle thickness was evaluated both at rest and during unconscious activation
Azadinia F. et al. (27)	2017	Iran	Orthosis Group: 20 (8/12, 27), Control Group: 21 (7/14, 27)	20–55	Non-extensible LSO (QuikDraw Brace) + routine physical therapy twice a week for 4 weeks	Routine physical therapy twice a week for 4 weeks	COP parameters, VAS, Oswestry Disability Index, Tampa Scale of Kinesiophobia
Hagiwara Y. et al. (28)	2017	Japan	Intervention Group: 54 (2/52, 44), Control Group: 53 (1/52, 44)	>20	Spinal Underwear for 3 months	No intervention Waiting list as control group	Subjective musculoskeletal symptoms, VAS, Athens Insomnia Scale, K6 Psychological Distress, RMDQ, Somatosensory Amplification Scale, Sagittal spinal alignment with spinal mouse, Shoulder, hip and spinal ROM
Ay Uslusoy, G. & Savaş (26)	2013	Türkiye	Extension-controlled LS: 35 (16/19, 41), Elastic LS: 36 (20/16, 40), Control Group: 36 (16/20, 37)	NR	Extension-controlled LS Elastic LS	NSAID	NPRS, Schober Test, RMDQ
Sato N. et al. (25)	2012	Japan	Intervention Group: 20, Control Group: 20, Total Sample Size: 40 (20/20)	30–78	Lumbar canvas corset for 6 months + NSAID	NSAID	Japanese Orthopaedic Association (JOA Score) Biering-Sorenson Test, myoelectrical activities muscle fatigue %MPF
Yoshitaka Toda (24)	2002	Japan	Intervention Group: 72 (31/41, 41), Control Group: 71 (31/40, 39)	NR	Modified corset with front-to-back tension (worn during waking hours 4 weeks) + NSAID	Standard corset with back-to-front tension (worn during waking hours 4 weeks) + NSAID	Quebec Back Pain Disability Scale, Sacral Inclination Angle, Waist/Hip Ratio, Remission Rate

LSO, lumbo-sacral orthoses; LO, lumbar orthoses; LS, lumbar support; RMDQ, roland-morris disability questionnaire; VAS, visual analog scale; NPRS, numerical pain rating scale; NSAID, non-steroid anti-inflammatory drugs; ROM, range of motion; SC, standard care; NR, not reported.

**Table 2:** PICO framework for study selection.

Component	Criteria
**P** – Population	Adult individuals (≥18 years) with chronic low back pain (duration ≥12 weeks), including non-specific presentations and those with identifiable postural or structural subtypes (e.g., reduced lumbar lordosis, degenerative scoliosis). Participants with acute or subacute LBP, or post-surgical LBP, were excluded.
**I** – Intervention	Any type of trunk or lumbosacral orthosis (rigid, semi-rigid, soft, extensible, inextensible, corset, lumbar support, spinal brace), used alone or in combination with other conservative treatments (e.g., physiotherapy, exercise, pharmacotherapy).
**C** – Comparison	No orthotic intervention; physiotherapy or exercise alone; pharmacological treatment alone (e.g., NSAIDs); alternative orthotic designs; standard care.
**O** – Outcomes	Primary: pain intensity (e.g., VAS, NPRS); functional disability (e.g., Oswestry Disability Index, Roland-Morris Disability Questionnaire). Secondary: neuromuscular outcomes (e.g., EMG, muscle thickness via ultrasound, center of pressure); psychosocial outcomes (e.g., fear-avoidance, kinesiophobia); health-related quality of life; adverse effects; treatment adherence.
**S** – Study Design	Randomized controlled trials (RCTs) only.

The methodological quality of the included RCTs, assessed using the PEDro Scale, is presented in Table 3. Overall, PEDro scores ranged from 4/10 (“fair” quality) to 7/10 (“good” quality). Most trials reported adequate randomization and baseline comparability, but blinding of participants, therapists, and assessors was rarely achieved, and allocation concealment was inconsistently applied.

**Table 3:** Study of the methodological quality of the RCTs through the PEDro scale.

Author	1^a^	2	3	4	5	6	7	8	9	10	11	Total
de Oliveira FCL. et al. (23)	1	0	0	1	0	0	0	1	0	1	1	4/10 Fair
Hekmatfard M. et al. (33)	1	1	1	1	0	0	0	1	0	1	1	6/10 Good
Annaswamy TM. et al. (32)	1	1	0	1	0	0	0	1	0	1	1	5/10 Fair
Azadinia F. et al. (29)	1	1	1	1	0	0	0	1	0	1	1	6/10 Good
Samani M. et al. (31)	1	1	1	1	0	0	0	1	0	1	1	6/10 Good
Azadinia F. et al. (30)	1	1	1	1	0	0	1	1	0	1	1	7/10 Good
Azadinia F. et al. (27)	1	1	1	1	0	0	0	1	0	1	1	6/10 Good
Hagiwara Y. et al. (28)	1	1	0	0	0	0	1	1	1	1	1	6/10 Good
Ay Uslusoy, G. et al. (26)	1	1	0	1	0	0	0	1	0	1	1	5/10 Fair
Sato N. et al. (25)	1	1	1	1	0	0	0	0	0	1	0	4/10 Fair
Yoshitaka Toda (24)	1	0	0	1	0	0	0	1	0	1	1	4/10 Fair

The PEDro scale includes the following items: 1 – eligibility criteria specified (^a^ not included in the total score), 2 – random allocation, 3 – concealed allocation, 4 – baseline comparability, 5 – blinding of participants, 6 – blinding of therapists, 7 – blinding of assessors, 8 – adequate follow-up (>85% of participants), 9 – intention-to-treat analysis, 10 – between-group statistical comparisons, and 11 – reporting of point estimates with measures of variability. As Item 1 assesses external validity, it is not counted in the total score; therefore, the total PEDro score ranges from 0 to 10. In this study, scores ≤3 were considered “poor” quality, scores of 4–5 “fair” quality, scores of 6–8 “good” and scores ≥ 9 “excellent” quality.

Across the included studies, the total reported sample size was 691 participants. However, three studies by Azadinia F (27, 29, 30). appeared to involve the same participant cohort. After adjustment for this overlap, the total number of unique individuals included in the review was 606. Of these participants, 304 were allocated to intervention groups and 302 to control groups.

Of the 606 unique participants, 372 (∼61.4%) were female, and 234 (∼38.6%) were male. The study by Hagiwara Y (28). included a markedly higher proportion of female participants, whereas the remaining studies recruited both sexes with varying gender distributions. Participant ages ranged from 18 years (33) to 85 years (32). Based on the studies for which mean age data were available, the weighted mean age was calculated as 39.6 years. Mean age data could not be calculated for one study (25), as only the age range was reported.

All included studies focused on individuals with chronic non-specific low back pain. Lumbosacral orthoses (LSOs) were used as the main intervention in most of the included studies, with considerable variation in orthotic type and application protocols. These included semi-rigid inextensible LSOs (33), soft LSOs applied at normal and high pressure (31), and non-extensible LSOs (27, 29, 30). Other orthotic devices included the Horizon™ 627 lumbar orthosis (32), spinal underwear (28), and a lumbar canvas corset (25). One study compared two lumbar support designs, namely an extension-controlled lumbar support and an elastic lumbar support, with a control group (26). Adjunct or comparator treatments varied across studies and included NSAIDs, back school education, structured exercise programs, and routine physiotherapy, either as part of the intervention or control conditions.

The intervention duration across the included studies ranged from a single session to 12 months. De Oliveira et al. (23) assessed immediate effects after a single LSO application session. Most studies evaluated short-term effects after 3 to 12 weeks of orthosis use (24, 26, 27, 29–31, 33), whereas two studies assessed longer-term effects after 3 to 6 months of orthosis use or follow-up (25, 28).

### 3.2 Outcome measurements

Pain was the most commonly assessed outcome among the included studies. The Visual Analog Scale (VAS) (27, 28, 31) and the Numerical Pain Rating Scale (NPRS) (23, 26, 32) were the most frequently used tools to evaluate pain intensity. Additionally, other instruments such as the Japanese Orthopedic Association (JOA) Score (25) were also used in the one study. In four studies (23, 29, 30, 33), baseline pain levels were recorded; however, post-intervention pain outcomes were not reported.

Functional disability was another commonly evaluated outcome across the included studies. The Modified Oswestry Disability Questionnaire was used in one study (31) to assess the degree of functional impairment associated with low back pain, while the Oswestry Disability Index was used in one study (27). The Roland-Morris Disability Questionnaire (RMDQ), utilized in two studies (26, 28), served to evaluate physical disability related to daily activities. One study (32) employed the EuroQol 5-Dimension (EQ-5D) to measure health-related quality of life. Additionally, the Quebec Back Pain Disability Scale was used in one study (24) to evaluate pain-related functional limitations in activities of daily living.

In addition to physical outcomes, some studies also assessed psychological factors related to chronic pain and disability. One study (27) employed the Tampa Scale for Kinesiophobia to assess the fear of movement or re-injury. These tools contributed to a broader understanding of how psychosocial variables may influence treatment outcomes.

In three studies (23, 27, 29), different COP parameters were utilized as outcome measurements. One of these studies (29) also examined postural conditions. Two studies (25, 31) employed EMG measurements to assess muscle activation and fatigue parameters, whereas one study (30) utilized ultrasound to measure muscle thickness.

### 3.3 The effectiveness of orthotic interventions

The effects of LSOs varied according to orthosis type, comparator intervention, co-interventions, and follow-up duration. De Oliveira et al. (23) assessed the immediate effect of an LSO after a single session and reported no superior effect on postural control compared with the abdominal drawing-in maneuver. In contrast, several studies evaluated short-term or longer follow-up effects. Toda (24) and Sato et al. (25) compared lumbosacral corset use combined with non-steroidal anti-inflammatory drugs (NSAIDs) with NSAID treatment alone, whereas Ay Uslusoy and Savaş (26) compared extension-controlled and elastic lumbar supports with NSAID treatment. Samani et al. (31) compared soft LSOs applied at different pressures with NSAID treatment. Overall, some studies reported improvements in pain or disability after LSO use, particularly in short-term intervention periods; however, the findings were heterogeneous and depended on the orthosis design, comparator, and outcome assessed.

Annaswamy et al. (32) reported that adding a one-size lumbar orthosis to a standardized exercise and education program resulted in worse outcomes in terms of disability and quality of life, compared to the same program delivered without an orthosis. The trial was terminated early due to these unfavorable outcomes.

Three studies by Azadinia and colleagues, which are believed to have included the same patient population, were included in this systematic review (27, 29, 30). In these studies, Azadinia et al. reported that adding a four-week non-extendible brace to physiotherapy had similar effects on postural sway and postural control as physiotherapy alone (27, 29), with only functional disability showing greater improvement in the group receiving both physiotherapy and bracing (27).

No findings were identified in the studies indicating that orthosis use leads to muscle atrophy. Samani et al. (31) reported that soft LSO use at different pressures did not have adverse effects on motor function or clinical parameters. Similarly, Sato et al. (25) found that six months of brace use did not increase muscle fatigue.

Azadinia et al. (30), who assessed the effect of orthosis use on deep trunk muscle morphology, found no significant changes in the thickness of the obliquus internus, transversus abdominis, or lumbar multifidus after four weeks of brace use combined with routine physiotherapy.

Toda (24) concluded that the use of an extendible brace, which provides physiological lumbar lordosis, was more effective in individuals with non-central obesity. He emphasized that the presence of central obesity and lumbar lordosis should be evaluated when prescribing brace treatment.

## 4 Discussion

The findings indicate that lumbosacral orthoses may improve pain and disability in some individuals with CLBP, mainly in the short term. However, the results were not consistent across studies. Differences in orthosis design, comparator interventions, co-interventions, and follow-up duration make it difficult to draw firm conclusions about their overall effectiveness.

Among the randomized controlled trials included in this review, pain and functional disability were the most frequently assessed outcomes. However, outcome reporting was not consistent across studies. Although pain intensity and disability were commonly evaluated using validated measures such as the VAS, NPRS, Oswestry-based questionnaires, and the Roland-Morris Disability Questionnaire (23, 26–28, 31, 32), objective neuromuscular outcomes were assessed in only a limited number of trials. These included EMG, center of pressure parameters, and ultrasound-based muscle thickness measurements (23, 25, 29–31). This limited use of objective measures restricts mechanistic insight into how orthoses may exert their effects.

Psychosocial variables were also infrequently assessed, with only Azadinia et al. (27) evaluating fear-avoidance beliefs or kinesiophobia. Given the biopsychosocial nature of CLBP, future trials should routinely include validated psychosocial measures, such as fear-avoidance, kinesiophobia, pain catastrophizing, self-efficacy, and treatment expectations, as these factors may influence patient selection, adherence, and response to orthotic interventions. Similarly, adherence to orthotic use was poorly reported, although it may be affected by discomfort, aesthetics, or reduced mobility. In addition, incomplete reporting of post-intervention pain outcomes in some studies limited the interpretation of within-group and between-group changes and weakened the overall synthesis of pain-related effects (23, 29, 30, 33). Future studies should therefore ensure complete reporting of both baseline and post-intervention outcomes to allow more robust comparisons across trials.

The included studies differed markedly in the type of orthosis used, wearing time, treatment duration, and accompanying interventions. Devices included soft, semi-rigid, extensible, and inextensible LSOs, as well as lumbar supports, spinal underwear, and lumbar canvas corsets (25, 27–33). Comparator or adjunct treatments also differed across studies and included NSAIDs, physiotherapy, kinesitherapy, back school education, and structured exercise programs (24–27, 31–33). These differences may partly explain the variability in outcomes and make direct comparison between studies difficult. Future studies should describe the orthosis type, wearing schedule, treatment duration, and co-interventions more clearly.

The inconsistent findings across studies suggest that the clinical effects of LSOs may depend on the comparator intervention, orthosis design, and treatment context. Some trials compared orthotic interventions with non-steroidal anti-inflammatory drugs (NSAIDs) or no orthotic intervention (24–26, 28, 31), whereas others used physiotherapy, exercise, education, or alternative orthotic designs as comparators (23, 27, 29, 30, 32, 33). Therefore, the observed effects should be interpreted cautiously because of heterogeneity in study design, comparator interventions, follow-up duration, and outcome reporting.

The absence of consistent benefits across all outcomes indicates that LSOs should not be considered uniformly effective for all individuals with CLBP. Instead, their clinical value may depend on patient selection, orthosis design, treatment goals, and integration with active rehabilitation. These findings suggest that LSO prescription in CLBP may be optimized through individual patient assessment rather than standardized prescription alone.

Patient-reported experiences with orthoses should also be considered when developing personalized treatment algorithms. Beyond biomechanical effects, some patients may perceive orthoses as providing security, reassurance, and support during daily activities. These subjective experiences may influence adherence, confidence in movement, patient satisfaction, and overall treatment response, and should therefore be systematically evaluated in future studies.

The response to LSO treatment may also depend on patient-related factors. Baseline muscle function, work-related demands, psychosocial status, body morphology, and patient preferences may all influence treatment response. However, these factors were rarely examined in the included studies. Only one study assessed kinesiophobia using the Tampa Scale for Kinesiophobia (27). Adverse effects such as discomfort, skin irritation, reduced mobility, and adherence problems were also inconsistently reported. Although trunk muscle deconditioning is a common concern, the available studies did not show orthosis-induced muscle atrophy or reduced muscle endurance (25, 30). Future trials should report adherence, tolerance, and adverse events more systematically.

Orthosis design and application context appeared to be important effect modifiers. High-pressure soft braces were associated with improved trunk strength and proprioception, while rigid orthoses provided greater spinal control (31). Despite these biomechanical changes, no significant improvements were observed in deep trunk muscle morphology. Moreover, orthotic interventions combined with physiotherapy or structured exercise consistently produced better results than orthosis use alone, reinforcing the importance of multimodal treatment approaches (25, 27). This supports the concept that orthoses should not be viewed as isolated interventions but rather as components of a comprehensive rehabilitation strategy.

When evaluating chronic low back pain in adults and considering corset prescription for treatment, the preservation of physiological lumbar lordosis and the presence of adult or degenerative scoliosis should be carefully assessed (13–15, 24). In individuals with reduced lumbar lordosis, sagittal realignment braces designed to restore lordosis, as well as high-correction, pattern-specific three-dimensional braces, can be effective in pain management and postural improvement, particularly in cases of adult scoliosis (13–15). In single lumbar or thoracolumbar curves, high-correction braces targeting structural curve reduction have the potential to improve both deformity and pain (14). Body morphology may also influence the response to LSO treatment. Toda (24) reported that lumbosacral extensible corsets improved functional outcomes in individuals without central obesity, whereas this benefit was not observed in individuals with central obesity. These findings suggest that orthotic prescriptions in CLBP should move beyond a “one-size-fits-all” approach and may be optimized by individual assessment of patients (24, 34). In addition, CLBP should not be regarded as a homogeneous condition. Weiss and Werkmann proposed a simple functional classification based on physical testing, suggesting that patients may be categorized into postural or instability-related subgroups, which could support more targeted conservative management and improve comparability across future studies (34). Although this evidence was derived from a case series rather than a randomized trial, it indicates that functional subclassification may be useful when interpreting orthotic indications and designing future studies (34).

Several methodological limitations were identified across the included RCTs. The interpretation of these findings should also consider the methodological limitations reflected in the PEDro scores. Although most trials reported random allocation and baseline comparability, participant and therapist blinding was rarely feasible, assessor blinding was inconsistently applied, and allocation concealment was not uniformly reported. These limitations may increase the risk of performance, detection, and selection bias, particularly because several outcomes, including pain intensity, disability, and perceived improvement, were subjective and patient-reported. Many studies had relatively small sample sizes, lacked detailed information on randomization or blinding procedures, and did not consistently report follow-up outcomes

Overall, the available evidence suggests that LSOs may be useful for some patients with CLBP, but their effects should not be generalized to all patients. The findings should be interpreted in light of differences in study quality, orthosis characteristics, comparator interventions, and follow-up duration. Future studies should use clearer patient-selection criteria, better-described orthosis protocols, and longer follow-up periods to identify which patients are most likely to benefit from LSO treatment.

## 5 Conclusion

This systematic review suggests that LSOs may provide short-term improvements in pain and disability in selected individuals with CLBP, particularly when integrated into multimodal rehabilitation programs. However, the findings should be interpreted with caution due to heterogeneity in orthosis design, intervention duration, comparator interventions, outcome measures, co-interventions, and patient characteristics. Evidence regarding longer-term effectiveness remains limited. Current evidence does not demonstrate orthosis-induced muscle atrophy or functional decline; however, adverse effects, adherence, patient tolerance, and patient-reported experiences were not consistently reported. Future high-quality RCTs with standardized protocols, complete outcome reporting, psychosocial measures, adverse-event monitoring, and longer-term follow-up are warranted to better define the role of LSOs in CLBP management.

## Data Availability

The raw data supporting the conclusions of this article will be made available by the authors, without undue reservation.
